# Hydroxypropyl Cellulose Enhances Immune Responses to the Current Seasonal Influenza Vaccine in Mice

**DOI:** 10.1111/1348-0421.70026

**Published:** 2025-12-01

**Authors:** Nantaporn Kaewaroon, Sara Yoshimoto, Luthfi Muawan, Shintaro Shichinohe, Tokiko Watanabe

**Affiliations:** ^1^ Department of Molecular Virology, Research Institute for Microbial Disease The University of Osaka Suita Osaka Japan; ^2^ National Vaccine Institute, Mueang Nonthaburi Nonthaburi Thailand; ^3^ Center for Infectious Disease Education and Research (CiDER) The University of Osaka Suita Osaka Japan; ^4^ Center for Advanced Modalities and DDS (CAMaD) The University of Osaka Suita Osaka Japan

**Keywords:** adjuvant, hydroxypropyl cellulose, seasonal influenza vaccine

## Abstract

Influenza A and B viruses cause annual epidemics and continue to pose global public health concerns. The most effective approach to preventing or mitigating the severity of influenza is vaccination. Inactivated split influenza HA vaccines are commonly used worldwide due to their strong safety profile and broad range of target groups; however, their efficacy is suboptimal, especially in the elderly. Adjuvants are used to enhance the effectiveness of some influenza vaccines, but few adjuvants have been approved for human vaccines. Previously, we identified hydroxypropyl cellulose as a promising adjuvant for the split influenza HA vaccine for the 2015–2016 and 2016–2017 influenza seasons. Here, we evaluated whether hydroxypropyl cellulose could enhance the efficacy of the quadrivalent split HA vaccine for the 2023–2024 influenza season, which contains the HA proteins of A/Victoria/4897/2022 (H1N1)pdm09 and three other strains. Using a mouse model, we performed immunogenicity studies and assessed protective efficacy against challenges with homologous and heterologous H1N1pdm09 virus strains. We found that hydroxypropyl cellulose in combination with the HA vaccine generated higher virus‐specific IgG antibody titers compared to the vaccine alone. The adjuvanted vaccine provided complete protection against homologous challenge and enhanced viral clearance from respiratory organs. Notably, the adjuvanted vaccine demonstrated cross‐protective efficacy against heterologous H1N1pdm09 virus challenge, improving survival rates compared to vaccine alone. Our results demonstrate that hydroxypropyl cellulose has potential as an adjuvant for current seasonal influenza vaccines.

**Abbreviations**
Alum: aluminum hydroxide gel
ANOVA: analysis of variance
AUC: area under the curve
BSA: bovine serum albumin
BSL2: Biosafety level 2
DPI: day post‐infection
ELISA: enzyme‐linked immunosorbent assay
GISAID: global initiative on sharing all influenza data
HA: hemagglutinin
HAU: hemagglutination units
HI: hemagglutination Inhibition
MA‐Cal04: mouse‐adapted A/California/04/2009 (H1N1)pdm09
MDCK: Madin‐Darby canine kidney
MEM: minimum essential medium
MLD_50_
: 50% mouse lethal dose
NA: neuraminidase
OD: optical density
PBS: phosphate buffered saline
PFU: plaque‐forming units
RBCs: red blood cells
RDE: receptor destroying enzyme
SD: standard deviation

## 1 Introduction

Seasonal influenza is an acute respiratory infection caused by influenza A and B viruses. The A/H1N1, A/H3N2, B/Victoria lineage, and B/Yamagata lineage viruses cause annual epidemics and raise serious global public health concerns. There are an estimated one billion cases of seasonal influenza each year, with 3–5 million of those cases resulting in severe illness and 290,000–650,000 respiratory fatalities [1]. Vaccination is the most efficient approach to prevent or reduce the severity of influenza. Three main types of influenza vaccine are available: inactivated (egg‐ or cell‐derived), recombinant, and live attenuated. Inactivated split influenza HA vaccines are widely used due to their broad range of target populations and robust safety profile [2, 3]; however, their effectiveness is suboptimal, especially in those over 65 years of age who are considered as high‐risk. The challenge of developing effective influenza vaccines is further complicated by the continuous antigenic evolution of influenza viruses, particularly via their hemagglutinin (HA) protein.

Antigenic changes and mutations in HA can induce viral escape from human neutralizing immunity [4, 5]. The strains for the seasonal influenza vaccine are recommended by the World Health Organization (WHO) based on the current dominant circulating strains, and mismatches due to such genetic changes in HA can result in reduced vaccine efficacy. Since the A/H1N1pdm09 virus emerged and caused a pandemic in 2009, H1H1pdm09 viruses have undergone continuous evolution of both their HA and neuraminidase (NA) segments, with particularly accelerated diversification after 2017 [5, 6, 7, 8]. These antigenic changes in HA and NA have led to the diversity of influenza viruses, generating new clades and subclades. We previously analyzed the HA segments of influenza A(H1N1)pdm09 viruses isolated between 2016 and 2019 and report on the diversity of the virus clades [6]. Our extended phylogenetic analysis of H1N1pdm09 strains isolated between 2009 and 2024 has revealed substantial divergence in the HA segment, with particularly pronounced differentiation observed during epidemic periods after 2017, resulting in the emergence of multiple distinct clades and subclades.

Adjuvants can be used to increase the efficacy of influenza vaccines [9], yet few adjuvants are licensed for use in human vaccines. Only one adjuvant‐containing vaccine (MF59) [10], has been authorized and licensed for use in the United States and is only approved for use in those aged 65 and older [9, 10, 11]. Several potential adjuvants for seasonal influenza vaccines are being evaluated [12]. Previously, we used an animal model to assess injectable excipients as adjuvants for the split influenza HA vaccine for the 2015–2016 and 2016–2017 season [13]. Among the candidates, hydroxypropyl cellulose showed superior enhancement of antibody responses and survival rates compared to aluminum hydroxide gel (alum). However, the vaccine used in this previous study contained A/California/7/2009 HA protein, which belongs to Clade 1, whereas currently circulating strains have evolved and now belong to subclade 6B.1 A.5a.2a.1, as a represented by A/Victoria/4897/2022. Given the substantial antigenic divergence of these clades and the ongoing evolution of H1N1pdm09 viruses, it is essential to evaluate whether hydroxypropyl cellulose maintains its adjuvanticity with current vaccine formulations.

Here, we examined the adjuvant potential of hydroxypropyl cellulose by using the current quadrivalent split HA vaccine for the 2023–2024 influenza season, which contains the HA proteins of A/Victoria/4897/2022 (H1N1)pdm09, A/Darwin/9/2021 (H3N2), B/Austria/1359417/2021 (Victoria lineage), and B/Phuket/3073/2013 (Yamagata lineage) [14]. This vaccine composition is relevant as three of its four components are included in both the current seasonal influenza vaccine (2024–2025) [15] and the upcoming vaccine (2025–2026) [16]. Using a mouse model, we performed immunogenicity studies and assessed protective efficacy and viral shedding in organs using both a homologous H1N1pdm09 virus (A/Victoria/4897/2022) and a heterologous mouse‐adapted H1N1pdm09 virus (MA‐Cal04). Our findings demonstrate the efficacy of hydroxypropyl cellulose as an adjuvant for current seasonal influenza vaccines and its potential to enhance cross‐protective immunity against antigenically distinct viral strains.

## 2 Materials and Methods

### 2.1 Cells and Viruses

Madin‐Darby canine kidney (MDCK) cells were maintained in minimum essential medium (MEM) supplemented with 5% newborn calf serum (Sigma), penicillin, and streptomycin at 37°C in 5% CO₂. MDCK cells were used for plaque assays to determine virus titers for challenge inoculation and organ titration.

Influenza virus A/Victoria/4897/2022 (IVR‐238) (H1N1)pdm09, whose HA protein is a component of the seasonal influenza split HA vaccine for the 2023–2024 influenza season, was kindly provided by the National Institute of Infectious Diseases (NIID), Japan, and used for the homologous challenge study. Mouse‐adapted A/California/04/2009 (H1N1)pdm09 (MA‐Cal04) [17], was kindly provided by Professor Yoshihiro Kawaoka and used for the heterologous challenge study. Both virus strains were propagated in the allantoic cavity of 10‐day‐old embryonated chicken eggs at 37°C in 45% humidity for 48 h. Allantoic fluid was harvested, aliquoted, and titrated by plaque assay. Virus stocks were stored at −80°C until use. All viral experiments were performed under biosafety level 2 (BSL2) conditions using wild‐type isolates and recombinant viruses approved by the Institutional Review Board of the Research Institute for Microbial Diseases, The University of Osaka (protocol number: BIKEN‐ 00225‐020). MA‐Cal04 is a recombinant virus generated by reverse genetics and approved for use by the Ministry of Education, Culture, Sports, Science and Technology (approval number: 4767‐1).

### 2.2 Phylogenetic Tree Analysis

For the phylogenetic analysis, we selected 73 A(H1N1)pdm09 strains isolated between 2009 and 2024 (Supporting Information S1: Table S1). These strains included WHO‐recommended vaccine strains for each season in the Northern and Southern Hemispheres [6]. Phylogenetic analysis was performed using HA gene sequences obtained from the GISAID EpiFlu database (https://gisaid.org) with the neighbor‐joining method [18] by using Kimura distances [19]. Bootstrap analysis was conducted with 1000 replicates to assess the reliability of the tree topology. All phylogenetic analyses were performed using Molecular Evolutionary Genetic Analysis (MEGA) software (version 11.0.13) [20, 21]. All sequences were aligned by using the MUSCLE (MUltiple Sequence Comparison by Log‐Expectation) algorithm [22].

### 2.3 Influenza Vaccine and Adjuvants

The quadrivalent seasonal influenza split HA vaccine for the 2023–2024 season was kindly provided by the Research Foundation for Microbial Diseases of Osaka University, Japan. The vaccine contained 30 μg/mL of HA protein from each of the following four strains: A/Victoria/4897/2022 (IVR‐238) (H1N1)pdm09, A/Darwin/9/2021 (SAN‐010) (H3N2), B/Austria/1359417/2021 (BVR‐26) (Victoria lineage), and B/Phuket/3073/2013 (Yamagata lineage). The vaccine was diluted in phosphate buffered saline (PBS; without calcium or magnesium) (Nacalai Tesque Inc.), and vaccine doses were calculated based on the HA content of A/Victoria/4897/2022 (IVR‐238) (H1N1)pdm09.

Aluminum hydroxide gel (Alhydrogel adjuvant 2%, InvivoGen) was used as a positive control adjuvant. The vaccine and alum were mixed at a 1:1 (v/v) ratio, resulting in a final concentration of 500 μg of alum per dose. The mixture was thoroughly mixed by pipetting for at least 5 min to allow antigen adsorption to the adjuvant.

Hydroxypropyl cellulose was suspended in PBS (without calcium or magnesium) at 10 mg/mL and prepared as previously described [13, 23]. Briefly, the suspension was sonicated in a water bath for 15 min and stored at −25°C until use. Before mixing with the vaccine, the adjuvant stock was thawed at room temperature and then sonicated for 5 min. The final concentration of hydroxypropyl cellulose in the vaccine formulation was 100 μg per dose.

### 2.4 Immunization and Challenge of Mice

All animal experiments were conducted in accordance with the guidelines of the Research Institute for Microbial Diseases, The University of Osaka, and approved by the Institutional Animal Care and Use Committee of the Research Institute for Microbial Diseases, The University of Osaka (approval number: R04‐04‐0).

Six‐week‐old female BALB/c mice (Japan SLC Inc.) were immunized intramuscularly twice at a 2‐week interval with 100 μL per dose; PBS, alum alone, or hydroxypropyl cellulose alone served as negative controls. Vaccine groups received the optimal dose of 0.0003 μg per dose of split HA vaccine alone, or vaccine combined with hydroxypropyl cellulose or alum. Two weeks after the second immunization, serum samples were collected for determination of virus‐specific antibody titers by use of ELISA and HI assays.

Three weeks after the second immunization, immunized mice were challenged with 2 × 10^5^ Plaque‐Forming Units (PFU) of A/Victoria/4897/2022 (IVR‐238) for the homologous challenge or 10 MLD_50_ (the 50% mouse lethal dose) of MA‐Cal04 (equivalent to 4.68 × 10^4^ PFU) for the heterologous challenge via intranasal inoculation of 50 μL/mouse (25 μL per nostril). Following challenge, body weight and survival were monitored daily for 14 days in a subset of mice (*n* = 4 per group). Mice that lost more than 25% of their initial body weight were humanely euthanized. Body weight changes were calculated as the mean ± SD, and percent survival was analyzed using GraphPad Prism version 9.5.1.

To assess viral replication and clearance in the respiratory tract, nasal turbinate and lungs were collected from the challenged mice at 3 and 6 days post‐infection. These organs were homogenized, and virus titers were determined by plaque assay using MDCK cells. Viral titers are expressed as log_10_PFU per gram of tissue.

### 2.5 Hemagglutination Inhibition (HI) Assay

Sera collected from immunized mice were treated with receptor‐destroying enzyme [RDE(II); DENKA SEIKEN] to remove nonspecific inhibitors overnight at 37°C, followed by heat inactivation at 56°C for 30 min. The treated sera were diluted 10‐fold with PBS and then serially diluted two‐fold in 96‐well plates.

Twenty‐five microliters of diluted sera were mixed with an equal volume of 8 hemagglutination units (HAU) of virus antigen and incubated at room temperature for 30 min. Fifty microliters of 1% guinea pig RBCs were added and incubated at room temperature for 1 h. HI titers are expressed as the reciprocals of the highest serum dilution that completely inhibited hemagglutination [24, 25].

### 2.6 Enzyme‐Linked Immunosorbent Assay

Virus‐specific IgG antibody titers in mouse sera were measured by using a modified ELISA as previously described [13, 26]. For antigen preparation, virus particles were pelleted by ultracentrifugation, resuspended, and subjected to sucrose gradient purification. Fractions with the highest hemagglutination activity were pooled, and protein concentrations were determined using the Micro BCA Assay kit (Thermo Scientific). The purified virus was inactivated and used as the coating antigen.

Ninety‐six‐well plates (Thermo Scientific) were coated with 50 μL of 0.5 μg/mL inactivated purified virus and incubated overnight at 4°C. Plates were washed with PBS containing 0.05% Tween‐20 (PBS‐T) and blocked with 200 μL per well of 5 mg/mL bovine serum albumin (BSA; Fraction V, protease‐free, Roche) overnight at 4°C. After the wells were washed once with PBS‐T, 50 μL of two‐fold serially diluted serum samples in PBS was added to each well and incubated at room temperature for 2 h.

Bound IgG antibodies were detected by incubation with horseradish peroxidase (HRP)‐conjugated goat anti‐mouse IgG (diluted 1:10000 in PBS; 50 μL per well) at room temperature for 2 h. After the plates were washed with PBS‐T, 100 μL of o‐phenylenediamine dihydrochloride substrate (FUJIFILM Wako) was added to each well. The reaction was stopped after 10 min, and optical density (OD) was measured at 492 nm (OD_492_). The OD₄₉₂ values were analyzed, and the area under the curve (AUC) was calculated to determine the level of virus specific IgG antibody in the mouse sera. Data were visualized by using GraphPad Prism version 9.5.1.

### 2.7 Virus Titration in Organs

To determine virus titers in respiratory organs, nasal turbinate and lungs collected from challenged mice were stored at −80°C until processing. Organ samples were thawed at room temperature, weighed, and transferred to 2‐mL tubes containing 1 mL of MEM supplemented with BSA (1 × MEM/BSA) and magnetic beads. Samples were homogenized twice using a TissueLyser III (QIAGEN) under the condition of 30 Hz frequency for 1 min each time. Tissue debris was pelleted by centrifugation at 8000 rpm for 5 min at 4°C, and supernatants were collected for virus titration.

Virus titers were determined by plaque assay on MDCK cells. Briefly, organ homogenates were serially diluted 10‐fold, and plaques were counted after incubation. Virus titers were calculated as PFU per mL and expressed as log₁₀ PFU per gram of tissue after dividing by the organ weight.

### 2.8 Statistical Analysis

Data were analyzed by using GraphPad Prism version 9.5.1. Statistical comparisons of ELISA data (AUC), HI titers, and virus titers in respiratory organs were performed between the vaccine alone group and the vaccine plus adjuvant groups (alum or hydroxypropyl cellulose) using one‐way ANOVA followed by Tukey's multiple comparison test. Survival rates were analyzed using simple survival analysis (Kaplan‐Meier) and comparing the survival curves. *p* values were calculated with the log‐rank (Mantel‐Cox) test.

## 3 Results

### 3.1 Phylogenetic Analysis of A(H1N1)pdm09 Viruses

To assess the phylogenetic relationship and evolutionary divergence of A(H1N1)pdm09 viruses, we analyzed 73 virus strains (Supporting Information S1: Table S1) isolated between 2009 and 2024, including A/Victoria/4897/2022, which represents the H1N1 component of the 2023–2024 seasonal vaccine used in this study, and A/California/7/2009, which we used in our previous adjuvant evaluation study [6].

**Figure 1:**
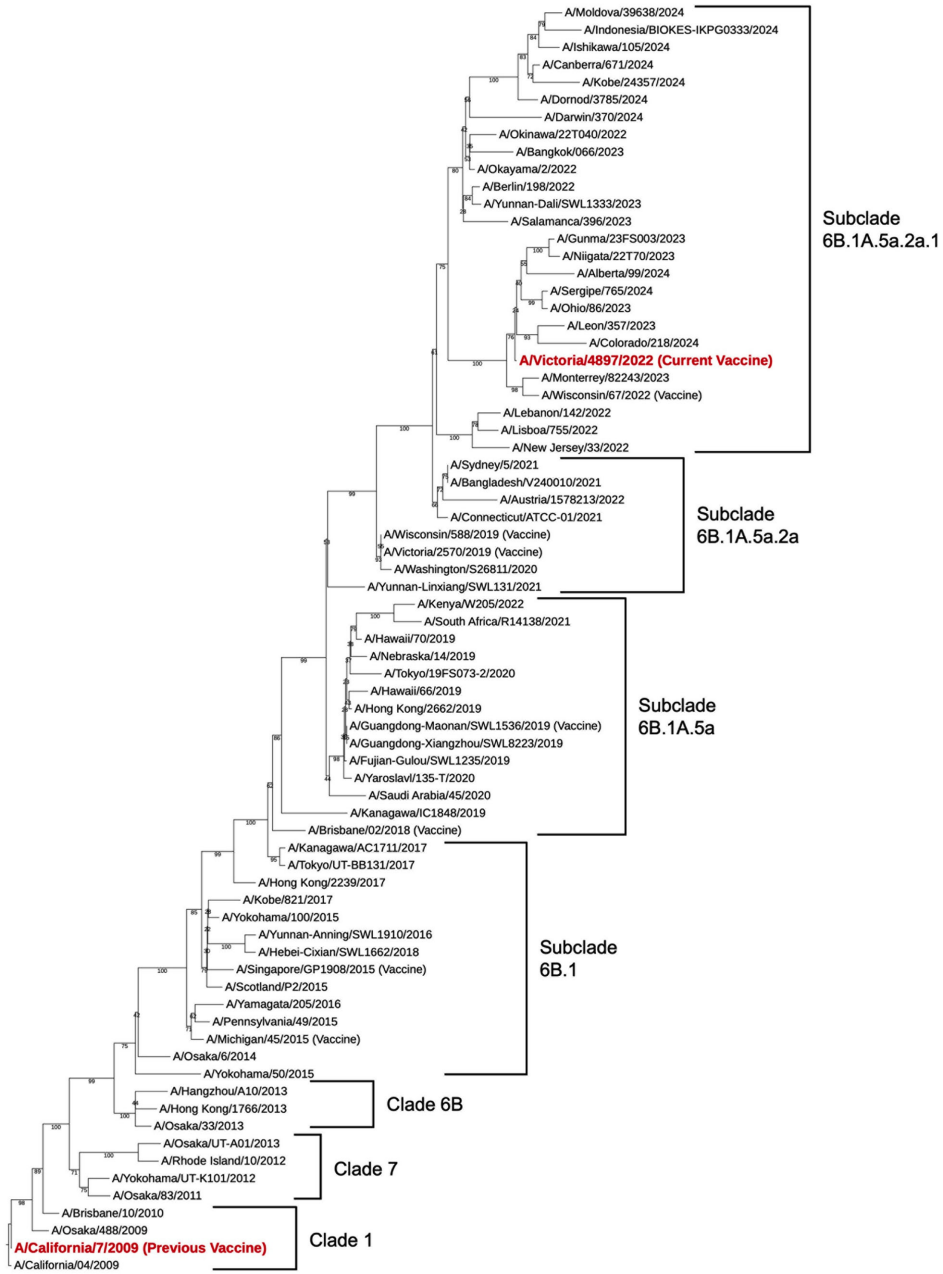
Phylogenetic analysis of A(H1N1)pdm09 viruses based on HA nucleotide sequences. The phylogenetic tree was constructed using the neighbor‐joining method with 73 virus strains isolated between 2009 and 2024. Viral clades and subclades are indicated by brackets. The vaccine strain used in this study (A/Victoria/4897/2022, subclade 6B.1 A.5a.2a.1) and the vaccine strain from our previous study (A/California/7/2009, Clade 1) are indicated in red. The tree demonstrates substantial antigenic divergence, particularly after 2017. The complete list of viral strains and WHO‐recommended vaccine strains is provided in Supporting Information S1: Table S1.

The phylogenetic analysis revealed seven major clusters among the 73 virus strains (Figure 1). Substantial genetic divergence in the HA segment from the ancestral Cal04/2009 strain was evident. Notably, Clade 6B.1 has diversified into multiple distinct subclades, including 6B.1 A.5a, 6B.1 A.5a.2a, and 6B.1 A.5a.2a.1. The vaccine strain A/Victoria/4897/2022 (used in this study) belongs to subclade 6B.1 A.5a.2a.1, whereas the vaccine strain used in our previous adjuvant evaluation study (A/California/7/2009) belongs to Clade 1. A/California/7/2009 was used as the WHO‐recommended vaccine strain from the 2010 to 2011 season to the 2016 to 2017 season, but was replaced in 2017 to 2018 due to a drift in antigenicity [27, 28, 29]. Given the substantial genetic distance between these vaccine strains and previous reports of accelerated antigenic diversification in H1N1pdm09 viruses since 2017 [4, 5, 6, 7, 8, 30], we recognized the need to re‐evaluate the adjuvant efficacy of hydroxypropyl cellulose with the current vaccine formulation.

### 3.2 Hydroxypropyl Cellulose Enhances Antibody Responses to the Current Seasonal H1N1 Vaccine Strain

To access the adjuvant potential of hydroxypropyl cellulose for the current seasonal influenza vaccine, we immunized BALB/c mice intramuscularly twice with a 2‐week interval. Six‐week‐old female mice received PBS, alum, or hydroxypropyl cellulose alone as negative controls, or the optimal vaccine dose (0.0003 μg) alone or combined with hydroxypropyl cellulose or alum. Two weeks after the second immunization, sera were collected and analyzed for virus‐specific IgG antibody responses against A/Victoria/4897/2022 (IVR‐238) by use of an ELISA and a HI assay.

**Figure 2:**
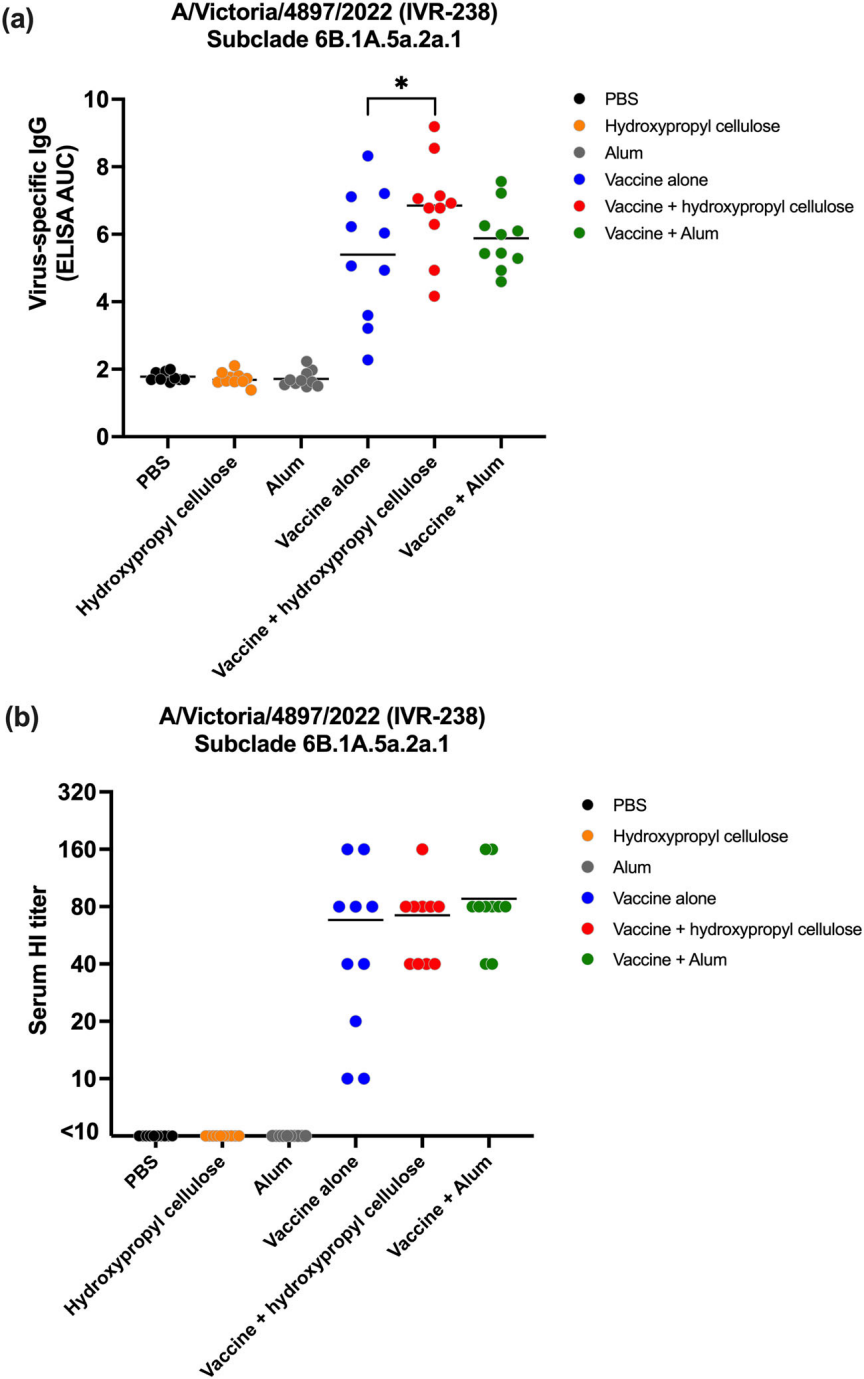
Virus‐specific antibody titers in mice against homologous strain A/Victoria/4897/2022 (IVR‐238). To evaluate virus‐specific antibody titers, six‐week‐old BALB/c mice were immunized twice intramuscularly with PBS, alum, or hydroxypropyl cellulose as negative controls. For the vaccine groups, mice were immunized with 0.0003 μg (optimum dose) per dose of vaccine alone, or vaccine plus hydroxypropyl cellulose or plus alum. Two weeks after the second vaccination, blood was collected, and ELISA and HI tests were used to determine specific antibody levels. (a) The area under the curve analyzed from the OD results at 492 nm by ELISA determines the level of virus‐specific antibody (IgG) in the mouse serum. (b) Serum HI titers using 1% guinea pig RBCs. The data were visualized by using GraphPad Prism version 9.5.1. Statistical analysis was performed using a one‐way ANOVA followed by Tukey's multiple comparison test. An asterisk indicates a significant difference between the vaccine alone group and the vaccine + hydroxypropyl cellulose group, *p < 0.05.

All vaccine groups, with or without adjuvant, induced substantial virus‐specific IgG antibody titers against A/Victoria/4897/2022 (IVR‐238) compared to the negative controls (Figure 2a). The vaccine adjuvanted with hydroxypropyl cellulose produced significantly higher IgG titers than vaccine alone (*p* < 0.05), with levels comparable to those achieved with vaccine plus alum. Similarly, HI assay results demonstrated that both adjuvant‐containing vaccine formulations induced higher HI titers compared to vaccine alone (Figure 2b), although the differences were not statistically significant. All negative controls (PBS, alum alone, and hydroxypropyl cellulose alone) showed minimal HI titers, which were below the detection limit (< 10).

These results demonstrate that hydroxypropyl cellulose can enhance humoral immune responses to the current seasonal H1N1 vaccine strain, with efficacy comparable to that of alum.

### 3.3 Hydroxypropyl Cellulose‐Adjuvanted Vaccine Provides Complete Protection Against Homologous H1N1 Challenge

To evaluate protective efficacy, immunized mice (*n* = 4 per group) were challenged intranasally with 2 × 10^5^ PFU of A/Victoria/4897/2022 (IVR‐238) 3 weeks after the second immunization. Body weight and survival rates were monitored daily for 14 days post‐challenge.

**Figure 3:**
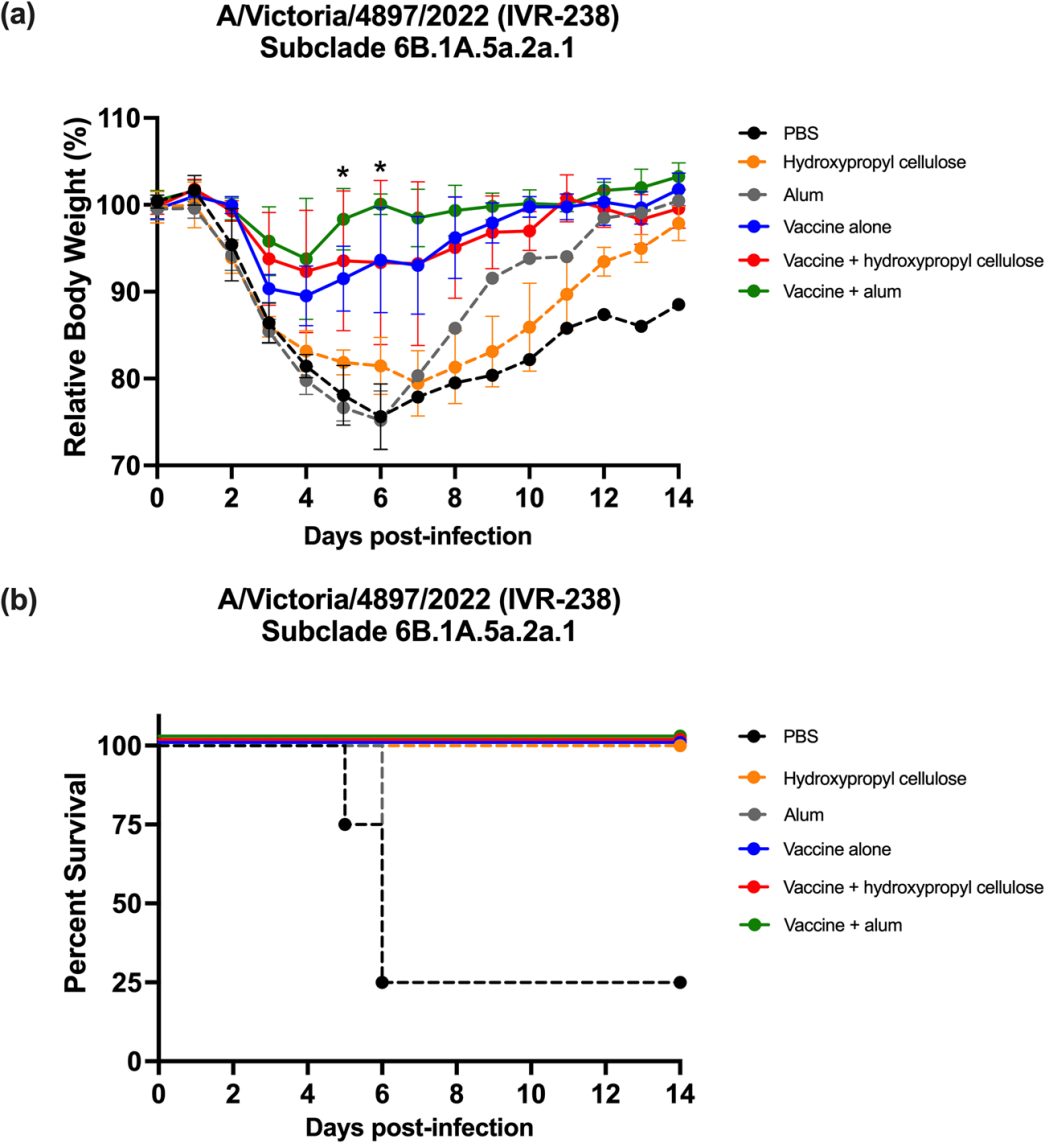
Body weight change and survival rate after homologous strain challenge. Three weeks after the second immunization, mice (n = 4 per group) were challenged with 2 × 10^5^ PFU per head of A/Victoria/4897/2022 (IVR‐238). Fifty microliters were inoculated intranasally (25 μL/nostril) under anesthesia. (a) After inoculation, individual body weights were measured, and relative body weight changes are presented as the mean ± SD. (b) The survival of mice in each group was recorded and the percent survival was calculated. The data were analyzed and plotted using GraphPad Prism version 9.5.1. Statistical analysis for relative body weight changes (mean ± SD) were performed using a one‐way ANOVA followed by Tukey's multiple comparison test. Asterisks indicate a significant difference between the vaccine alone group and the vaccine + alum group, *p < 0.05. Survival rates were analyzed by using simple survival analysis (Kaplan‐Meier) and comparing the survival curves. *p* values were calculated with the log‐rank (Mantel‐Cox) test.

All vaccinated groups, regardless of adjuvant inclusion, showed 100% survival following homologous virus challenge, which was significantly different from the negative control groups, which exhibited 75% mortality (*p* < 0.01) (Figure 3b). Although all vaccine groups achieved complete protection, the adjuvanted vaccine formulations led to less body weight loss compared to that of mice immunized with the vaccine alone (Figure 3a). The vaccine plus hydroxypropyl cellulose group showed an average maximum body weight loss of 7.68%, which was lower than that of the vaccine alone group (10.48%) but slightly higher than that of the vaccine plus alum group (6.23%). In contrast, the negative control groups experienced severe body weight loss of greater than 20% (PBS, 24.38%; alum alone, 24.81%; hydroxypropyl cellulose alone, 20.54%).

These results demonstrate that the split HA vaccine provides complete protection against homologous virus challenge and that adjuvants contribute to reduced morbidity as evidenced by attenuated body weight loss.

### 3.4 Enhanced Viral Clearance in Respiratory Organs With Adjuvanted Vaccines

To assess viral replication and clearance in the respiratory tract after virus challenge, nasal turbinate and lungs were collected from challenged mice (*n* = 3 per group) at 3 and 6 days post‐infection, and viral titers were determined by use of plaque assays.

At 3 days post‐infection, mice immunized with adjuvanted vaccines showed significantly lower viral titers in both the nasal turbinate and lungs compared to the negative control and vaccine alone groups (Table 1). The vaccine plus hydroxypropyl cellulose group exhibited viral titers of 3.9 ± 0.2 log₁₀ PFU/g in nasal turbinate, whereas the lung viral titers were reduced or undetectable in some mice. Similarly, the vaccine plus alum group showed viral titers of 3.5 ± 0.5 log₁₀ PFU/g in the nasal turbinate and no detectable virus in the lungs. In contrast, the vaccine alone group showed viral titers of 4.9 ± 0.2 log₁₀ PFU/g in the nasal turbinate and 6.4 ± 0.2 log₁₀ PFU/g in the lungs. Notably, mice with HI titers ≥ 80 showed no detectable virus in the lungs at 3 days post‐infection (Supporting Information S1: Table S2), suggesting a correlation between HI antibody levels and protection against viral replication in the lungs.

**Table 1:** Virus titers in the respiratory tract of immunized mice after challenge with homologous A/Victoria/4897/2022 (IVR‐238) virus^a^.

Immunogen	Virus titer (mean log_10_PFU ± SD/g) in:			
	Nasal turbinate		Lung	
	3 dpi	6 dpi	3 dpi	6 dpi
PBS	5.6 ± 0.3	4.6 ± 0.6	6.8 ± 0.1	5.8 ± 0.6
Hydroxypropyl cellulose	5.4 ± 0.2	4.0 ± 0.8	7.0 ± 0.3	5.0 ± 0.4
Alum	5.5 ± 0.3	3.8 ± 0.9	6.8 ± 0.0	4.4 ± 0.2
Vaccine alone	4.9 ± 0.2	3.2, 2.0, ND^b^	6.4 ± 0.2	3.9, ND, ND
Vaccine + hydroxypropyl cellulose	3.9 ± 0.2**	ND, ND, ND	4.5, ND, 4.9*	ND, ND, ND
Vaccine + alum	3.5 ± 0.5**	ND, ND, ND	ND, ND, ND	ND, ND, ND

^a^Six‐week‐old mice (*n* = 3 per group) were immunized with immunogen via intramuscular injection twice with a two‐week interval. Mice were inoculated with 2 × 10^5^ PFU per head of A/Victoria/4897/2022 (IVR‐238) at three weeks after the second immunization. Organs, including nasal turbinate and lung, were collected at 3 and 6 days post‐infection (dpi). The organs were homogenized, and the virus titer was measured by plaque assay using the MDCK cell line. Statistical analysis using a one‐way ANOVA followed by Tukey's multiple comparison test showed significant differences compared with the vaccine alone group: *p < 0.05, **p < 0.01. ^b^ND, not detectable.

By 6 days post‐infection, both adjuvanted vaccine groups (hydroxypropyl cellulose and alum) achieved complete viral clearance, with no detectable virus in either the nasal turbinate or lungs (Table 1). In contrast, the vaccine alone group still showed residual viral titers in some mice, although the levels were reduced compared to those at 3 days post‐infection. The negative control groups maintained high viral titers throughout the observation period.

These results demonstrate that adjuvanted vaccines significantly enhance viral clearance from respiratory organs, with complete elimination of detectable virus by 6 days post‐infection, and this enhanced protection correlated with higher HI titers (i.e., ≥ 80).

### 3.5 Adjuvanted Vaccines Provide Partial Cross‐Protection Against Heterologous H1N1 Challenge

To investigate cross‐protective immunity, we assessed antibody responses, protective efficacy, and viral clearance following challenge with the heterologous MA‐Cal04 strain. Sera collected 2 weeks after the second immunization were analyzed by use of an ELISA and an HI assay for cross‐reactive antibodies against MA‐Cal04.

**Figure 4:**
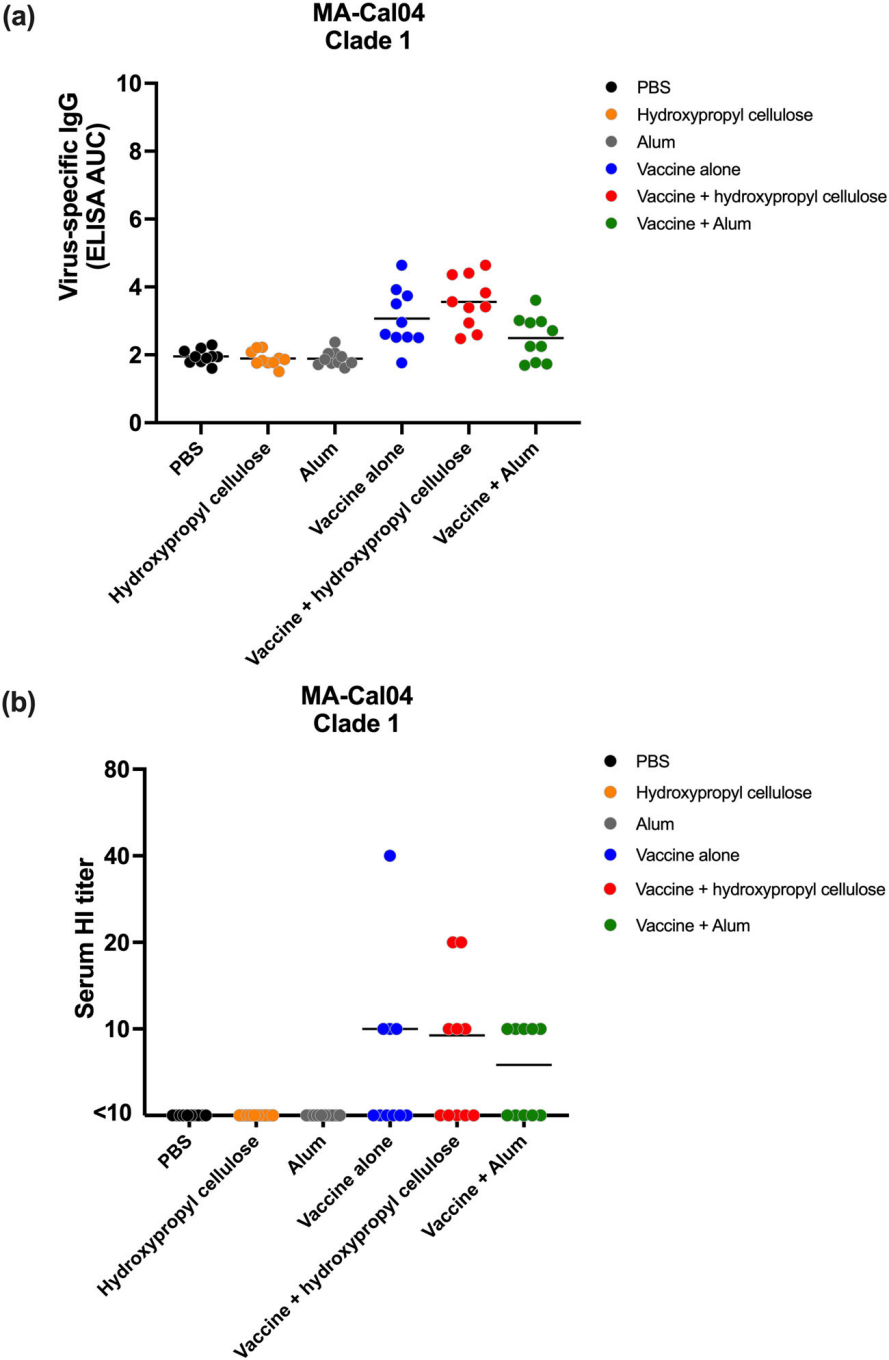
Virus‐specific antibody titers against heterologous strain MA‐Cal04. To assess cross‐immunogenicity in mice, six‐week‐old BALB/c mice (*n* = 4 per group) were immunized with split HA vaccine twice intramuscularly with PBS, alum, or hydroxypropyl cellulose as negative controls. For the vaccine groups, mice were immunized with 0.0003 μg per dose (optimal dose) of vaccine alone, or vaccine plus hydroxypropyl cellulose, or plus alum. Two weeks after the second vaccination, blood was collected, and ELISA and HI tests were used to determine virus‐specific antibody titers. (a) The area under the curve analyzed from the OD results at 492 nm by ELISA determines the level of virus‐specific antibody (IgG) in the mouse serum. (b) Serum HI titers using 1% guinea pig RBCs. The data were analyzed and plotted using GraphPad Prism version 9.5.1. Statistical analysis was performed using a one‐way ANOVA followed by Tukey's multiple comparison test.

The ELISA results showed low levels of cross‐reactive IgG antibodies in the vaccinated groups, with the vaccine plus hydroxypropyl cellulose group showing slightly higher titers than the vaccine alone or vaccine plus alum groups, although the differences were not statistically significant (Figure 4a). Similarly, the HI assay revealed minimal neutralizing activity against the heterologous strain, with titers comparable to those of the negative controls (Figure 4b).

**Figure 5:**
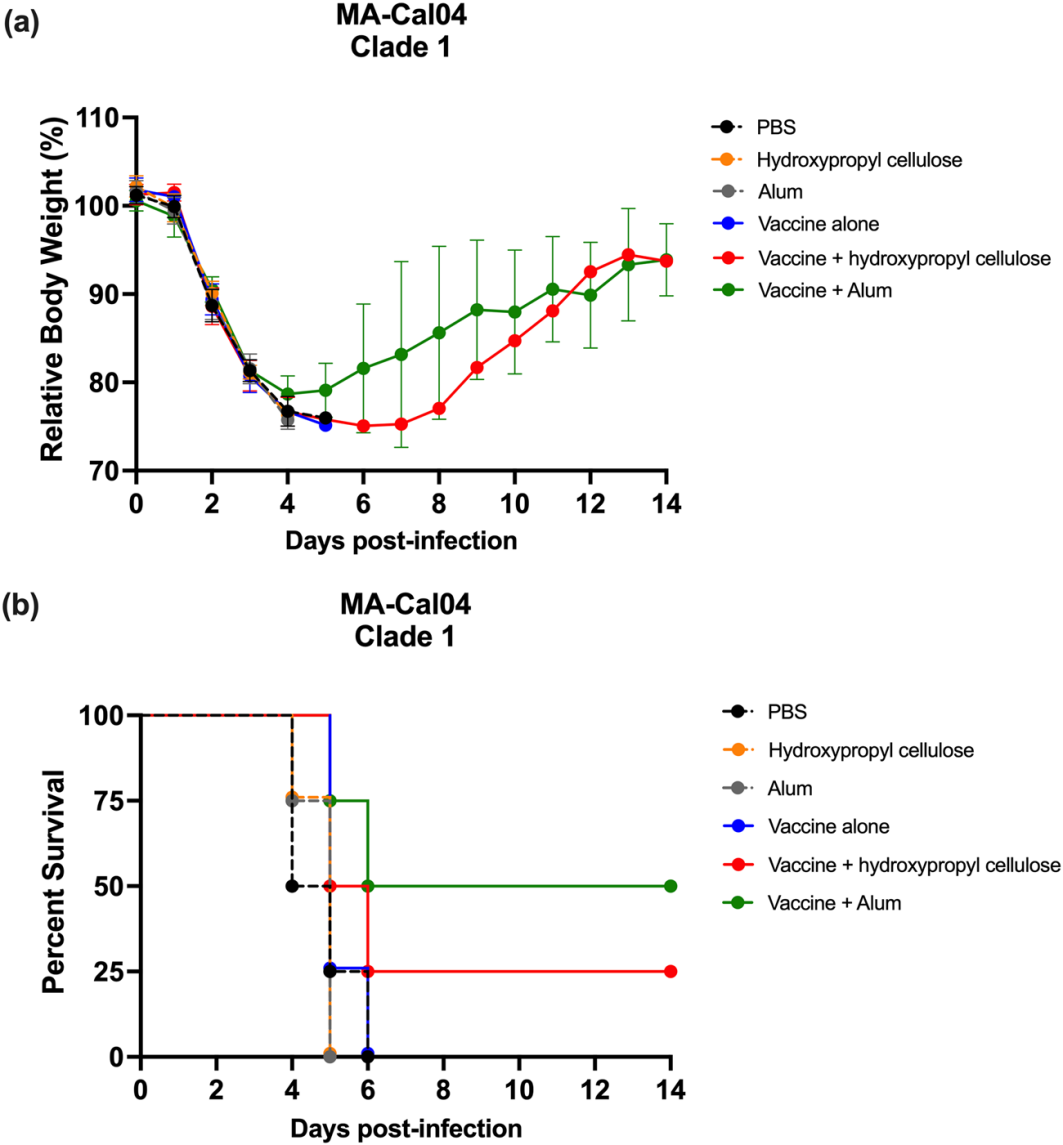
Body weight change and survival after heterologous strain challenge. Three weeks after the second immunization, mice were challenged with 10 MLD_50_ (equivalent to 4.68 × 10^4^ PFU per head) of MA‐Cal04. Fifty microliters were inoculated intranasally (25 μL/nostril) under anesthesia. (a) After inoculation, individual body weights were measured, and the relative body weight changes are presented as the mean ± SD. (b) The survival of mice in each group was recorded and the percent survival was calculated. The data were analyzed and plotted using GraphPad Prism version 9.5.1. Statistical analysis for relative body weight changes (mean ± SD) were performed using a one‐way ANOVA followed by Tukey's multiple comparison test. Survival rates were analyzed by using simple survival analysis (Kaplan‐Meier) and comparing the survival curves. *p* values were calculated with the log‐rank (Mantel‐Cox) test.

Despite the low cross‐reactive antibody titers, challenge with 10 MLD_50_ of heterologous MA‐Cal04 1 week after serum collection revealed differences in protective efficacy among groups. All groups experienced similar levels of body weight loss (exceeding 20%), with no significant difference between the vaccine and negative control groups (Figure 5a). However, the mice that received the adjuvanted vaccines showed improved survival rates: 50% for the vaccine plus alum group and 25% for the vaccine plus hydroxypropyl cellulose group, compared to 0% survival for the negative control and vaccine alone groups (Figure 5b).

Analysis of viral titers in respiratory organs showed high viral loads in all groups at both 3 and 6 days post‐infection (Table 2). At 3 days post‐infection, viral titers ranged from 7.1 to 7.8 log₁₀ PFU/g in the nasal turbinate and 7.8–8.1 log₁₀ PFU/g in the lungs across all groups, with no significant differences. By 6 days post‐infection, viral titers remained elevated in all groups, including those that received adjuvanted vaccines, indicating limited viral clearance capacity against the heterologous strain.

**Table 2:** Virus titers in the respiratory tract of immunized mice after challenge with heterologous mouse‐adapted A/California/04/2009 virus^a^.

Immunogen	Virus titer (mean log_10_PFU ± SD/g) in:			
	Nasal turbinate		Lung	
	3 dpi	6 dpi	3 dpi	6 dpi
PBS	7.4 ± 0.2	6.1 ± 0.4	8.0 ± 0.2	7.4 ± 0.5
Hydroxypropyl cellulose	7.4 ± 0.3	6.9 ± 0.8	8.1 ± 0.2	6.6 ± 0.9
Alum	7.8 ± 0.2	6.4, 7.0, NA^b^	8.0 ± 0.3	7.7, 6.8, NA
Vaccine alone	7.3 ± 0.2	6.0 ± 0.1	7.9 ± 0.1	6.7 ± 0.5
Vaccine + hydroxypropyl cellulose	7.1 ± 0.1	5.8 ± 0.3	7.8 ± 0.2	6.9 ± 0.4
Vaccine + alum	7.2 ± 0.6	5.7 ± 0.5	8.0 ± 0.3	6.4 ± 0.4

^a^ Six‐week‐old mice (*n* = 3 per group) were immunized with immunogen via intramuscular injection twice with a two‐week interval. Mice were inoculated with 10 MLD_50_ (equivalent to 4.68 × 10^4^ PFU per head) of MA‐Cal04 at three weeks after the second immunization. Organs, including nasal turbinate and lung, were collected at 3 and 6 days post‐infection (dpi). The organs were homogenized, and the virus titer was measured by plaque assay using the MDCK cell line. Statistical analysis was performed using a one‐way ANOVA followed by Tukey's multiple comparison test. No significant differences were observed between vaccine alone and vaccine combined with adjuvants (*p* > 0.05).

^b^ NA, not applicable because the mouse died after blood collection

These results demonstrate that while the current H1N1 vaccine provides limited cross‐reactive antibody responses against heterologous strains, adjuvant supplementation can enhance cross‐reactive efficacy, improving survival rates despite high viral loads in respiratory organs.

## 4 Discussion

In this study, we evaluated the adjuvant potential of hydroxypropyl cellulose for the current seasonal influenza vaccine (2023–2024 season), which contains A/Victoria/4897/2022 (H1N1)pdm09. We found that hydroxypropyl cellulose combined with the split HA vaccine enhances humoral antibody responses, provides complete protection against homologous virus challenge, and improves viral clearance from respiratory organs. Previously, we identified hydroxypropyl cellulose as a promising adjuvant candidate for split influenza HA vaccine, showing superior efficacy compared to that of aluminum hydroxide gel (alum) [13]. However, that study used vaccines containing A/California/7/2009 (Clade 1) as the H1N1 component, whereas the current vaccine contains A/Victoria/4897/2022, which belongs to subclade 6B.1 A.5a.2a.1. There is substantial antigenic divergence between these clades, with particularly pronounced antigenic changes observed in H1N1pdm09 viruses after 2017 [5]. Since antigenic changes in HA can affect vaccine efficacy and may influence adjuvant performance, it was important to re‐evaluate hydroxypropyl cellulose with current vaccine formulations. Our results confirm that hydroxypropyl cellulose maintains its adjuvant efficacy with the current seasonal influenza vaccines despite the substantial antigenic evolution of H1N1pdm09 viruses.

Hydroxypropyl cellulose is a water‐soluble cellulose derivative that has been widely used as a pharmaceutical excipient; its functions including viscosity modification, emulsion stabilization, and enhancement of drug solubility and absorption [31, 32]. It has been approved for use in injectable formulations by regulatory authorities in Japan, the United States, Australia, and the European Union, and has a well‐established safety profile [33]. This regulatory status represents a major advantage for clinical translation, as the safety and tolerability of hydroxypropyl cellulose have already been established, potentially lowering the barriers to its clinical application as an adjuvant. Previous studies have reported that hydroxypropyl cellulose can stabilize antigens in freeze‐dried intranasal influenza vaccines and induce systemic IgG and mucosal IgA responses in mice [34]. The mechanism by which hydroxypropyl cellulose enhances immune responses as an adjuvant remains unclear, but several possibilities have been proposed, including the possibility that it may create a depot effect at the injection site, prolonging antigen exposure to immune cells. Moreover, its ability to enhance absorption across biological membranes suggests it could facilitate antigen uptake by antigen‐presenting cells. The induction of mucosal IgA reported in previous studies also suggests a potential role in enhancing mucosal immunity. However, the precise mechanism of its adjuvant activity warrants further investigation.

Our study has limitations that will be addressed in future research. First, we evaluated only a single dose and formulation of hydroxypropyl cellulose. Dose optimization studies, including evaluation of different vaccine doses and adjuvant‐to‐vaccine ratios, are needed to maximize efficacy. Second, our immunogenicity assessment focused on the H1N1 component, and the adjuvant effects on the H3N2 and influenza B components of the quadrivalent vaccine remain to be evaluated. In addition, we assessed only humoral immune responses, whereas evaluation of cell‐mediated immunity, including T cell responses, would provide a more comprehensive understanding of the immune mechanisms underlying the adjuvant effect. Finally, while we demonstrated that hydroxypropyl cellulose enhances vaccine efficacy, the precise mechanism underlying its adjuvant activity remains unclear and requires further investigation. Further studies addressing these limitations will strengthen the evidence in support of hydroxypropyl cellulose as a safe and effective adjuvant for seasonal influenza vaccines and may facilitate its clinical development.

## Author Contributions

Shintaro Shichinohe and Tokiko Watanabe conceptualized and designed the study. Nantaporn Kaewaroon, Sara Yoshimoto, Shintaro Shichinohe, and Tokiko Watanabe performed the experiments. Luthfi Muawan performed the phylogenetic tree analysis. Nantaporn Kaewaroon, Sara Yoshimoto, Luthfi Muawan, Shintaro Shichinohe, and Tokiko Watanabe analyzed the data. Nantaporn Kaewaroon and Tokiko Watanabe wrote the original draft. Sara Yoshimoto, Luthfi Muawan, and Shintaro Shichinohe reviewed and edited the manuscript. Tokiko Watanabe supervised the project. All authors have read and agreed to the final version of the manuscript.

## Conflicts of Interest

The authors declare no conflicts of interest.

## Ethics Statement

This study was approved by the Institutional Animal Care and Use Committee of the Research Institute for Microbial Diseases, The University of Osaka (approval number: R04‐04‐0).

## Supporting information


**Supplementary Table 1.** List of A(H1N1)pdm09 viruses used in this study. **Supplementary Table 2.** HI titers of immunized mice and organ titers after challenge with homologous A/Victoria/4897/2022 (IVR‐238) virus.

## Data Availability

The data that support the findings of this study are available from the corresponding author upon reasonable request.
