# Supplementary material for: Hydroxypropyl Cellulose Enhances Immune Responses to the Current Seasonal Influenza Vaccine in Mice
Source: Microbiol Immunol. 2025 Dec 1;70(2):68–79. doi: 10.1111/1348-0421.70026 (PMC12868939; doi:10.1111/1348-0421.70026)
Supplement: Supplementary file 1 — Supplementary Table 1. List of A(H1N1)pdm09 viruses used in this study. Supplementary Table 2. HI titers of immunized mice and organ titers after challenge with homologous A/Victoria/4897/2022 (IVR‐238) virus. [file MIM-70-68-s001.docx]

**Supplementary information**

**Hydroxypropyl cellulose enhances immune responses to the current seasonal influenza vaccine in mice**

Nantaporn Kaewaroon, Sara Yoshimoto, Luthfi Muawan, Shintaro Shichinohe, and Tokiko Watanabe

- **Supplementary Tables 1-2**

**Supplementary Table 1. List of A(H1N1)pdm09 viruses used in this study.**

| **No.** | **Virus name** | **Isolate ID** | **Clade** | **Vaccine Season *** |
| --- | --- | --- | --- | --- |
| 1 | A/Osaka/488/2009 | EPI_ISL_393908 | 1 |  |
| 2 | A/California/7/2009 * | EPI_ISL_391380 | 1 | 2010–2011 to 2016–2017 |
| 3 | A/California/04/2009 | EPI_ISL_393964 | 1 |  |
| 4 | A/Brisbane/10/2010 | EPI_ISL_218721 | 1 |  |
| 5 | A/Osaka/83/2011 | EPI_ISL_393947 | 7 |  |
| 6 | A/Rhode Island/10/2012 | EPI_ISL_17559613 | 7 |  |
| 7 | A/Yokohama/UT-K101/2012 | EPI_ISL_393909 | 7 |  |
| 8 | A/Osaka/UT-A01/2013 | EPI_ISL_393910 | 7 |  |
| 9 | A/Hangzhou/A10/2013 | EPI_ISL_148646 | 6B |  |
| 10 | A/Hong Kong/1766/2013 | EPI_ISL_144513 | 6B |  |
| 11 | A/Osaka/33/2013 | EPI_ISL_393948 | 6B |  |
| 12 | A/Osaka/6/2014 | EPI_ISL_393950 | 6B.1 |  |
| 13 | A/Pennsylvania/49/2015 | EPI_ISL_205493 | 6B.1 |  |
| 14 | A/Yokohama/100/2015 | EPI_ISL_202312 | 6B.1 |  |
| 15 | A/Yokohama/50/2015 | EPI_ISL_174814 | 6B.1 |  |
| 16 | A/Scotland/P2/2015 | EPI_ISL_207910 | 6B.1 |  |
| 17 | A/Singapore/GP1908/2015 * | EPI_ISL_236220 | 6B.1 | 2017–2018 to 2018–2019, Cell-based |
| 18 | A/Michigan/45/2015 * | EPI_ISL_336680 | 6B.1 | 2017–2018 to 2018–2019, Egg-based |
| 19 | A/Yunnan-Anning/SWL1910/2016 | EPI_ISL_242526 | 6B.1 |  |
| 20 | A/Yamagata/205/2016 | EPI_ISL_240015 | 6B.1 |  |
| 21 | A/Hong Kong/2239/2017 | EPI_ISL_268711 | 6B.1 |  |
| 22 | A/Kobe/821/2017 | EPI_ISL_266846 | 6B.1 |  |
| 23 | A/Kanagawa/AC1711/2017 | EPI_ISL_292812 | 6B.1 |  |
| 24 | A/Tokyo/UT-BB131/2017 | In-House Data | 6B.1 |  |
| 25 | A/Hebei-Cixian/SWL1662/2018 | EPI_ISL_367318 | 6B.1 |  |
| 26 | A/Brisbane/02/2018 * | EPI_ISL_406712 | 6B.1A.5a | 2019–2020 |
| 27 | A/Fujian-Gulou/SWL1235/2019 | EPI_ISL_367295 | 6B.1A.5a |  |
| 28 | A/Guangdong-Maonan/SWL1536/2019 * | EPI_ISL_377081 | 6B.1A.5a | 2020–2021 |
| 29 | A/Hong Kong/2662/2019 | EPI_ISL_377246 | 6B.1A.5a |  |
| 30 | A/Guangdong-Xiangzhou/SWL8223/2019 | EPI_ISL_367275 | 6B.1A.5a |  |
| 31 | A/Nebraska/14/2019 | EPI_ISL_397255 | 6B.1A.5a |  |
| 32 | A/Hawaii/66/2019 | EPI_ISL_397020 | 6B.1A.5a |  |
| 33 | A/Kanagawa/IC1848/2019 | EPI_ISL_394984 | 6B.1A.5a |  |
| 34 | A/Hawaii/70/2019 | EPI_ISL_397028 | 6B.1A.5a |  |
| 35 | A/Yaroslavl/135-T/2020 | EPI_ISL_19824860 | 6B.1A.5a |  |
| 36 | A/Saudi Arabia/45/2020 | EPI_ISL_18037996 | 6B.1A.5a |  |
| 37 | A/Tokyo/19FS073_2/2020 | EPI_ISL_16196398 | 6B.1A.5a |  |
| 38 | A/South Africa/R14138/2021 | EPI_ISL_18385058 | 6B.1A.5a |  |
| 39 | A/Kenya/W205/2022 | EPI_ISL_19822006 | 6B.1A.5a |  |
| 40 | A/Wisconsin/588/2019 * | EPI_ISL_404527 | 6B.1A.5a.2a | 2021–2022 to 2022–2023, Cell-based |
| 41 | A/Victoria/2570/2019 * | EPI_ISL_15907696 | 6B.1A.5a.2a | 2021–2022 to 2022–2023, Egg-based |
| 42 | A/Washington/S26811/2020 | EPI_ISL_19606048 | 6B.1A.5a.2a |  |
| 43 | A/Bangladesh/V240010/2021 | EPI_ISL_20135217 | 6B.1A.5a.2a |  |
| 44 | A/Yunnan-Linxiang/SWL131/2021 | EPI_ISL_20111814 | 6B.1A.5a.2a |  |
| 45 | A/Sydney/5/2021 | EPI_ISL_18879344 | 6B.1A.5a.2a |  |
| 46 | A/Connecticut/ATCC-01/2021 | EPI_ISL_18291259 | 6B.1A.5a.2a |  |
| 47 | A/Austria/1578213/2022 | EPI_ISL_17820366 | 6B.1A.5a.2a |  |
| 48 | A/New Jersey/33/2022 | EPI_ISL_18274870 | 6B.1A.5a.2a.1 |  |
| 49 | A/Okayama/2/2022 | EPI_ISL_17244487 | 6B.1A.5a.2a.1 |  |
| 50 | A/Lisboa/755/2022 | EPI_ISL_17063107 | 6B.1A.5a.2a.1 |  |
| 51 | A/Berlin/198/2022 | EPI_ISL_16894916 | 6B.1A.5a.2a.1 |  |
| 52 | A/Okinawa/22T040/2022 | EPI_ISL_18077877 | 6B.1A.5a.2a.1 |  |
| 53 | A/Lebanon/142/2022 | EPI_ISL_17981162 | 6B.1A.5a.2a.1 |  |
| 54 | A/Victoria/4897/2022 * | EPI_ISL_19192400 | 6B.1A.5a.2a.1 | 2023–2024, Egg-based |
| 55 | A/Wisconsin/67/2022 * | EPI_ISL_19440421 | 6B.1A.5a.2a.1 | 2023–2024, Cell-based |
| 56 | A/Gunma/23FS003/2023 | EPI_ISL_20195340 | 6B.1A.5a.2a.1 |  |
| 57 | A/Niigata/22T70/2023 | EPI_ISL_20182597 | 6B.1A.5a.2a.1 |  |
| 58 | A/Bangkok/066/2023 | EPI_ISL_20144660 | 6B.1A.5a.2a.1 |  |
| 59 | A/Yunnan-Dali/SWL1333/2023 | EPI_ISL_20111916 | 6B.1A.5a.2a.1 |  |
| 60 | A/Ohio/86/2023 | EPI_ISL_19609093 | 6B.1A.5a.2a.1 |  |
| 61 | A/Salamanca/396/2023 | EPI_ISL_20090305 | 6B.1A.5a.2a.1 |  |
| 62 | A/Leon/357/2023 | EPI_ISL_20090268 | 6B.1A.5a.2a.1 |  |
| 63 | A/Monterrey/82243/2023 | EPI_ISL_19784215 | 6B.1A.5a.2a.1 |  |
| 64 | A/Ishikawa/105/2024 | EPI_ISL_20190074 | 6B.1A.5a.2a.1 |  |
| 65 | A/Kobe/24357/2024 | EPI_ISL_20193009 | 6B.1A.5a.2a.1 |  |
| 66 | A/Indonesia/BIOKES-IKPG0333/2024 | EPI_ISL_20125563 | 6B.1A.5a.2a.1 |  |
| 67 | A/Colorado/218/2024 | EPI_ISL_20077101 | 6B.1A.5a.2a.1 |  |
| 68 | A/Alberta/99/2024 | EPI_ISL_20067620 | 6B.1A.5a.2a.1 |  |
| 69 | A/Dornod/3785/2024 | EPI_ISL_20190019 | 6B.1A.5a.2a.1 |  |
| 70 | A/Darwin/370/2024 | EPI_ISL_20157858 | 6B.1A.5a.2a.1 |  |
| 71 | A/Sergipe/765/2024 | EPI_ISL_20144413 | 6B.1A.5a.2a.1 |  |
| 72 | A/Canberra/671/2024 | EPI_ISL_19871638 | 6B.1A.5a.2a.1 |  |
| 73 | A/Moldova/39638/2024 | EPI_ISL_19858919 | 6B.1A.5a.2a.1 |  |

* WHO (World Health Organization) recommended influenza virus vaccine strains.

| **Supplementary Table 2.**  **HI titers of immunized mice and organ titers after challenge with homologous A/Victoria/4897/2022 (IVR-238) virus ^a^** | | | | | | |
| --- | --- | --- | --- | --- | --- | --- |
| Immunogen | HI titer against A/Victoria | Virus titer (log_10_PFU±SD/g) in: | | | | |
|  |  | Nasal turbinate | |  | Lung | |
|  |  | 3 dpi | 6 dpi |  | 3 dpi | 6 dpi |
| PBS | <10, <10, <10 | 5.8, 5.5, 5.3 | - |  | 6.8, 6.9, 6.7 | - |
|  | <10, <10, <10 | - | 4.7, 5.2, 3.9 |  | - | 5.5, 6.4, 5.4 |
| Alum | <10, <10, <10 | 5.4, 5.4, 5.8 | - |  | 6.8, 6.8, 6.8 | - |
|  | <10, <10, <10 | - | 2.9, 3.8, 4.7 |  | - | 4.3, 4.3, 4.7 |
| Hydroxypropyl cellulose | <10, <10, <10 | 5.5, 5.2, 5.5 | - |  | 7.1, 7.3, 6.7 | - |
|  | <10, <10, <10 | - | 4.9, 3.3, 3.7 |  | - | 5.5, 4.7, 4.9 |
| Vaccine alone | 10, 10, 10 | 4.7, 5.1, 4.9 | - |  | 6.6, 6.1, 6.4 | - |
|  | 20, 10, 20 | - | 3.2, 2.0, ND^b^ |  | - | 3.9, ND, ND |
| Vaccine + alum | 80, 80, 80 | 4.0, 3.5, 3.1 | - |  | ND, ND, ND | - |
|  | 80, 80, 80 | - | ND, ND, ND |  | - | ND, ND, ND |
| Vaccine + Hydroxypropyl cellulose | 40, 80, 40 | 4.0, 3.7, 3.8 | - |  | 4.5, ND, 4.9 | - |
|  | 160, 40, 80 | - | ND, ND, ND |  | - | ND, ND, ND |
| ^a^ Six-week-old mice (n=3 per group) were immunized with the indicated immunogen via intramuscular injection twice with a two-week interval. Mice were inoculated with 2 x 10^5^ PFU per head of A/Victoria (H1N1) at three weeks after the second immunization. Organs, including nasal turbinate and lung, were collected at 3 and 6 days post-infection (dpi). The organs were homogenised, and the virus titers weew measured by plaque assay using the MDCK cells.  ^b^ ND, not detectable | | | | | | |
